# 

**Published:** 1911-01-28

**Authors:** 


### THE HOSPITAL

Telegraphic Address: Ospedale, London.

THE MODERN MEDICAL AND INSTITUTIONAL JOURNAL.

Telephone Number: 2734 Gerrard.

NEW SERIES. No. 208, Vol. VIII. No. 1280, Vol. XLIX.

SATURDAY, JAN. 28TH, 1911.

PRICE THREEPENCE

